# Bispecific Sigma1R-Antagonist/MOR-Agonist Compounds for Pain

**DOI:** 10.7759/cureus.59837

**Published:** 2024-05-07

**Authors:** Robert B Raffa, Joseph V Pergolizzi

**Affiliations:** 1 Pharmacology, Temple University, Philadelphia, USA; 2 Pain Management, NEMA Research, Naples, USA

**Keywords:** analgesic, mu-opioid receptor (mor), bispecific receptor affinity, s1r subtype, sigma receptors

## Abstract

Recent research has significantly advanced an understanding of sigma receptors, which consist of two distinct subtypes designated as S1R and S2R (*s1R* and *s2R* gene products, respectively). Both subtypes have recently been cloned and their crystal structures have been published. As a result, highly selective S1R and S2R agonist and antagonist ligands are now available. Unlike the confusion generated from prior use of non-selective ‘sigma’ compounds, these tool compounds have begun to add clarity about the function of sigma receptors in health and disease.

The discovery of compounds with high-affinity (nM range) S1R/S2R or S2R/S1R subtype selectivity (>100-fold), and selectivity over off-target sites (>1,000-fold) has brought the study of sigma receptor pharmacology into the modern era. Computer modeling has contributed to a better understanding of the binding processes, structural requirements for chemical synthesis, and potential therapeutic uses. Several lines of evidence converge on pain as a therapeutic target for S1R-antagonists (as single mechanism or as part of a multi-mechanistic approach). We highlight here some compounds reported over the past few years that have promise for use as analgesics, specifically some mono-mechanistic S1R-antagonists, and some that are ‘bispecific’, i.e., have more than one mechanism of action, for example, complementary action of the mu-opioid receptor (MOR). We concentrate on some compounds that are further along in development, in particular, some of the bispecific S1R-antagonist/MOR-agonist compounds.

## Introduction and background

Sigma receptors have a long history of study, but clarity about their pharmacology and relevance for therapeutic utility has only recently been advanced after inadvertent detours and circuitous progress. In retrospect, there are reasons why early studies resulted in the perception that sigma receptors were a bit enigmatic. There was a notable lack of selective compounds, so the nature of the sigma portion of activity was obfuscated by the collateral (off-target) effects; the lack of a cloned receptor (for S1R or S2R, and a putative S3R); lack of a crystal structure (for directed discovery of selective compounds); and an atypical function (‘chaperone’), particularly for the S1R, which was not envisioned as a receptor type. Unlike the recognized traditional classes of receptor-mediated signal transduction, such as cell surface ligand-gated ion channels, G protein-coupled receptors (GPCRs), transmembrane receptor tyrosine kinases, and nuclear receptors, ligand-sensitive chaperone receptors are a fairly recent finding [1]. Chaperones assist in the attainment and maintenance of the fidelity required for the folding and transport of proteins to establish proper functioning. Such an activity would have broad beneficial therapeutic utility, particularly in situations of dysregulated or disrupted physiological pathways or excursions from baseline activity. One such therapeutic application is the treatment of pain.

As described in recent reviews a valuable role of sigma receptors is as mediators of pain-relieving mechanisms, and therefore they provide pharmacologic targets for the discovery and development of novel analgesic agents [2,3]. Conventional analgesic therapy and medications suffer from well-known challenges, such as insufficient clinical efficacy, worrisome adverse effects, or problems with abuse. Based on the publications summarized herein, recent developments of bispecific S1R-antagonist/MOR-agonist compounds suggest that they could provide efficacy and safety advantages over the current analgesic options.

Purpose

The analgesic activity of sigma ligands could portend the development of non-opioid agents, useful as stand-alone therapy or as adjunctive opioid-sparing therapy. In the last few years, selective S1R-antagonist compounds have been reported that display analgesic activity and, due to near specificity for sigma sites, a promising minimization of off-target adverse effects. One compound, a ‘bispecific’ (balanced dual action) S1R-antagonist plus MOR partial agonist has advanced to clinical trial. We present a short review of recent publications on the pharmacologic properties and analgesic activity of compounds that have this bispecific activity. 

## Review

Methods

A literature search was conducted using sources such as PubMed for publications in the English language (2004 - 2024) using search terms such as ‘sigma receptor’, ‘S1R’, ‘S2R’, ‘pain’, ‘analgesia’, 'antinociception', and combinations of them. In addition, citations within identified publications were searched. Review articles on the broad topics were used for background information. The material was reviewed, and assessed for its applicability to the topic of the review. Specifically, the source had to contain: identification of compounds that possess selective activity at the S1R, and evaluation of the analgesic activity of the compounds (used alone or in combination with compounds that have other analgesic mechanisms of action). 

Results

Antinociceptive Activity of Mono-Mechanistic S1R-Antagonists

A few of the recent publications on S1R antagonists are highlighted below to demonstrate various aspects of the antinociceptive activity of S1R compounds in animal models that are predictive of analgesic activity in humans ('antinociceptive' is used to indicate inhibition of response to a presumed painful or injurious stimulus in animals; in humans, 'analgesic' can be used when the sensation-perception can be expressed as 'pain'), and present significant in vivo data regarding separation between analgesic activity and adverse effects.

As an example, Kopp et al. found that in a series of 2,6 disubstituted tetrahydropyrans, the cyclohexyl methylamine (*2S*, *6R*)-3b compound had the highest affinity for S1R (*K*_i_ = 0.95 nM) [4]. Subcutaneous administration of the compound to mice produced an anti-allodynic effect (67% inhibition) in a capsaicin-induced mechanical allodynia model.

Romeo et al. designed, synthesized and evaluated a series of benzylpiperazine derivatives [5]. The compound (identified as 15 in the publication) displayed the highest S1R affinity (*K*_i_ = 1.6 nM) with nearly 900-fold selectivity over S2R. Administered i.p. to mice, it produced dose-related inhibition of formalin-induced inflammation and mechanical allodynia in the sciatic nerve constriction injury (CCI) model. It had no significant effect on mice in a rotarod assay (measure of sedation or impaired locomotor responses). 

Wilson et al. report the results of preclinical testing of the benzylpiperazine derivative compound SI-1/28 (1-(4-{(4-hydroxymethyl)phenyl)methyl}piperazin-1-yl)-5-phenylpentan-1-one oxalate) [6]. It has about 400-fold selective affinity for S1R over S2R (*K*_i_ = 6.1 nM vs 2,583 nM, respectively); affinity for off-target sites was not reported. SI-1/28 induced dose-dependent acute antinociceptive activity in mice (via the i.p. route) as assessed using the acetic acid abdominal constriction test (ED_50_ = 27.4 mg/kg). It also induced dose-dependent inhibition of formalin-induced licking (ED_50_ = 13.2 mg/kg, i.p.) and formalin-induced inflammatory pain in mice. A dose-dependent anti-allodynic (neuropathic pain) effect of SI-1/28 was demonstrated in the sciatic ligation constriction nerve injury (CCI) model following i.p. administration to mice. Its efficacy at the highest dose tested was about 75% the efficacy of gabapentin (i.p.). SI-1/28 was further evaluated for potential adverse effects. It produced (at 60 mg/kg, i.p.) no significant change in spontaneous ambulation or evoked locomotion, as well as no sedative effects. It also produced (at 60 mg/kg, i.p.) no significant effects on breathing rate, and displayed no place conditioning aversion or preference (an indication of drug ‘liking’), in accordance with the literature on S1R antagonists in general [7]. Self-administration studies were not conducted.

Allodynia is a characteristic of some neuropathic pain conditions in which normally non-painful stimuli are perceived as excruciatingly painful [8]. A subtype, mechanical-induced allodynia, induced by capsaicin is reduced in S1R knockout (KO) mice, and treatment of wild-type (WT) mice with S1R-antagonists yields an anti-allodynic effect that is countered by administration of S1R agonists [9]. The S1R-receptor antagonist S1RA (also E-52862) was superior to gabapentin in reducing allodynia in the partial sciatic nerve ligation model, and the analgesic activity of this compound was demonstrated in humans [9]. An antiallodynic effect of S1R antagonists of another chemical structural class (bicyclic tropane-based compounds with an exocyclic double bond) has also been reported by [10]. The *K*_i_ values at S1R ranged from 0.45 to 18 nM (but selectivity over S2R was only modest, 1.6- to 24-fold). At 32 mg/kg s.c., a tested compound was active against capsaicin-induced mechanical allodynia (measured using von Frey filaments) in mice. The anti-allodynic effect was completely reversed by prior administration of an S1R-agonist, strong evidence that the tested compound acted through S1R [10].

A rather atypical test of analgesic therapeutic potential was reported by Álvarez-Pérez et al. [11]. This study was the first to assess an S1R antagonist (BD1063) in two fibromyalgia-like mouse models. BD1063 (1-[2-ethyl]-4-methylpiperazine) is a selective S1R- antagonist (*K*_i _= 9 nM) with about 50-fold selective affinity over S2R. One model was subcutaneous (23-day) reserpine-induced myalgia (RIM6 mice), the other model was 23-day intramuscular acid saline solution injection (ASI) mice. The models consist of the emergence of reflexive and nonreflexive pain responses and other altered behavioral responses over 23 days. Repeated administration of BD1063 (i.p.) provided longer pain alleviation than did gabapentin, which is FDA-approved for the treatment of fibromyalgia syndrome. 

Mechanistic Hypotheses Regarding the Analgesic Action of S1R-Antagonists

Several standard mechanisms for S1R-antagonist-induced antinociception have been proposed [12]. Some less standard recent suggestions include the following.

Based on previous knowledge that: mice with a targeted deletion of the s1R gene do not develop neuropathy, mice that lack the binding protein gene Hint1 exhibit exaggerated allodynia, that S1R-antagonists are more effective against neuropathic pain of spinal origin when administered into intracerebral ventricles, and that neuropathic pain resulting from nerve injury involves the binding of alpha-2-delta-1 proteins to the NMDA (*N*-methyl-D-aspartate) subtype of glutamate receptors, Rodríguez-Muñoz et al. investigated the participation of S1Rs and HINT1 proteins in the formation of NMDA complexes within the periaqueductal gray (PAG), an important origin of descending pain-modulating neurons that impinge on afferent junctions in the spinal cord [13]. Chronic constriction injury (CCI) fails to promote the formation of alpha-2-delta-1-NMDA complexes in S1R KO (S1R­-/-) mice, and these mice do not develop allodynia. The findings of the study suggest that S1Rs promote the formation of NMDA complexes within the PAG, hence S1R antagonists should display anti-allodynic activity. 

Based on the known presence of the CC-chemokine ligand 2 (CCL2) in primary afferent fibers, and its role in the microglia-dependent neuronal activation in certain inflammatory pain, Chun et al. [14] evaluated the possibility that the peripheral antinociception mechanism of the S1R antagonist BD1047 (*N*'-[2-(3,4-dichlorophenyl)ethyl]-*N,N,N*'-trimethylethane-1,2-diamine) might involve attenuation of inflammation-invoked elevation of CCL2 in dorsal root ganglion (DRG) cells. Using the complete Freund’s adjuvant (CFA)-induced inflammation model in mice, they found that repeated administration of BD1047 significantly attenuated CCL2 immunoreactivity and microglial activation, and correspondingly attenuated both thermal- and mechanical hyperalgesia. The authors conclude that BD1047’s antinociceptive action is “substantially mediated” by inhibition of CCL2 release, which then attenuates the activation of spinal microglia that is associated with inflammatory pain.

Along similar lines, Choi et al. [15] reported that interleukin-1ß decreases the expression of S1R in astrocytes in mouse spinal cord during the induction phase of neuropathic pain. The neuropathic pain model was a modification of the standard chronic constriction injury (CCI) of the sciatic nerve. Following injury, S1R expression was increased in astrocytes in the ipsilateral spinal cord (not the contralateral side as control). IL-1ß suppressed the expression of the GluN1 subunit of the NMDA receptor and the development of mechanical allodynia in CCI mice. Administration of a S1R-agonist (PRE084, 2-morpholin-4-ylethyl 1-phenylcyclohexane-1-carboxylate) reversed the effect of IL-1B. Thus the anti-allodynic effect of an S1R-antagonist might involve a protective effect on the beneficial modulatory effect of IL-1ß. 

Bispecific S1R-Antagonists for Pain

There is a substantial literature that shows that a variety of known S1R-antagonists enhance the antinociceptive effect, and reduce the opioid adverse effect profile, of opioid analgesics [16]. Fu et al. recently provided additional evidence by reporting that a new S1R-antagonist that emerged from a structure-activity relationship study of 2,6-diazaspiro(3.4)octan-7-one derivatives (*K*_i_ = 14.5 nM, approximately 10-fold more selective for S1R than for S2R) enhanced morphine-induced antinociceptive effect in the hot-plate test (55^o^C) in mice, and antagonized the development of morphine analgesic tolerance in this test [17]. 

In recent years, several compounds that possess combined S1R-antagonist properties plus a contributing second pharmacologic mechanism of analgesic activity in the same molecule (termed ‘bispecific’ agents) have been reported [18, 19]. Some representatives of the most recent examples for demonstrative and comparative purposes include the following.

Xiong et al. reported the optimization, synthesis, and testing of a series of piperidinamide derivatives that possess bispecific S1R antagonist plus MOR-agonist activity [20]. The compound designated HKC-126 ((S)-*N*-(2-(4-benzylpiperidin-1-yl)propyl)-*N*-phenylpropionamide)) was selected for analgesic and adverse-effect testing compared to fentanyl. HKC-126 has similar affinity for S1R and MOR (*K*_i_ = 19.4 and 4.3 nM, respectively) and showed no significant binding to other sites (inhibition of selective reference ligands by 50% or more at 10 µM). Computer modeling displayed the docking pose with MOR. In the in vivo testing in mice and rats, HKC-126 displayed dose-dependent inhibition of acetic acid-induced response, formalin-induced behaviors, hot-plate test (55^o^C), and mechanical allodynia in the CCI model. Compared to equi-analgesic dose of fentanyl, HKC-126 produced less sedation and effect on respiration, and less signs of physical dependence and reward (abuse potential). With its acceptable pharmacokinetic (PK) profile, HKC-126 was suggested to have characteristics favorable for treating neuropathic pain.

Zhuang et al. reported the design, synthesis, in silico docking, and in vivo evaluation of a series of benzylamino-fentanyl derivatives in analgesic and adverse-effect tests [21]. Compound 68 (‘Tao-191’) displayed similar affinity for both S1R and MOR (*K*_i _= 36 and 6.5 nM) without significant binding to other sites (<50% inhibition at 1 µM of references) except for lower affinity for S2R (*K*_i_ = 448 nM). In the in vivo tests in mice and rats, ‘Tao-191’ (s.c.) produced dose-related antinociception in the acetic acid-induced abdominal constriction and formalin tests, and in a paclitaxel-induced neuropathic pain model. A particular highlight of this study was the demonstration of the in vivo contributions of both S1R and MOR mechanisms to the antinociceptive tests by the significant attenuation of Tao-191-induced antinociceptive effect by combined administration of the MOR-antagonist naloxone plus the selective S1R agonist PRE-084. In adverse-effects screens in mice or rats, Tao-191 had little effect compared to placebo on locomotor activity, constipation potential, motor coordination, respiration, naloxone-precipitated withdrawal, or conditioned place preference (indication of abuse potential). The authors suggest that the results provide a rationale for further exploration of bispecific S1R/MOR compounds for clinical use as effective analgesics. 

García et al. reported on a series of compounds resulting from modification of high-throughput screening (HTS) to yield piperazinyl cycloalkyl methyl propionamides that were shown to possess balanced bispecific S1R-antagonist and MOR-agonist activity [22]. The highlighted compound (18g) has modest, but similar affinity for S1R and MOR (*K*_i_ = 108 and 251 nM, respectively) without significant affinity for more than 150 other sites (receptors, transporters, ion channels, and enzymes) except for S2R (*K*_i_ = 413 nM). The compound (i.p.) displayed activity in the mouse paw-pressure test of acute pain and, following repeated administration over 10 days, an anti-allodynic (mechanical) effect in the partial sciatic nerve ligation (PSNL) model. Compared to oxycodone, it produced less inhibition of gastrointestinal transit of a charcoal meal (a surrogate of constipation) in a rodent model. 

Vidal-Torres et al. reported on WLB-73502, a bispecific S1R-antagonist + MOR-partial agonist compound. WLB-73502 has similar binding affinity for S1R and MOR (*K*_i_ = 118 and 64 nM, respectively) [23]. In vitro measures of efficacy (intrinsic activity) indicated an antagonist action at S1R and partial agonist action at MOR. The designation as an in vitro partial agonist was based on the ability of the irreversible MOR antagonist ß-funaltrexamine (ß-FNX, also termed ß-FNA) to dose-dependently reduce the maximum effect of WLB-73502 on MOR agonist-mediated inhibition of forskolin-stimulated cAMP production, whereas ß-FNA shifted the oxycodone dose-response curve to the left without much effect on oxycodone’s maximum effect. Regarding antinociceptive testing in rats, WLB-73502 (i.p.) dose-dependently inhibited paw pressure-induced mechanical nociception, radiant-heat tail-flick latency, and was active in a carrageenan-induced model of acute inflammatory pain and tactile allodynia (assessed by the response to pressure application by von Frey filaments), an osteoarthritis (OA) pain model (intra-knee joint injection of monosodium iodoacetate (MIA), and tactile allodynia in the spared nerve injury (SNI) model of neuropathic pain. Tolerance did not develop to the anti-allodynic effect after repeated twice-daily administration for 4 weeks. At full analgesic doses against allodynia in the OA model, WLB-73502 produced less inhibition of gastrointestinal transit and respiratory depression than did oxycodone, and emesis than did morphine. Plasma and brain levels (brain-to-plasma ratio = 2.57) and brain receptor occupancy (S1R = 57 - 63%, MOR = 50 - 51%) were also determined. The development of physical dependence, rewarding potential, and tolerance to reward were not evaluated. 

**Figure 1:**
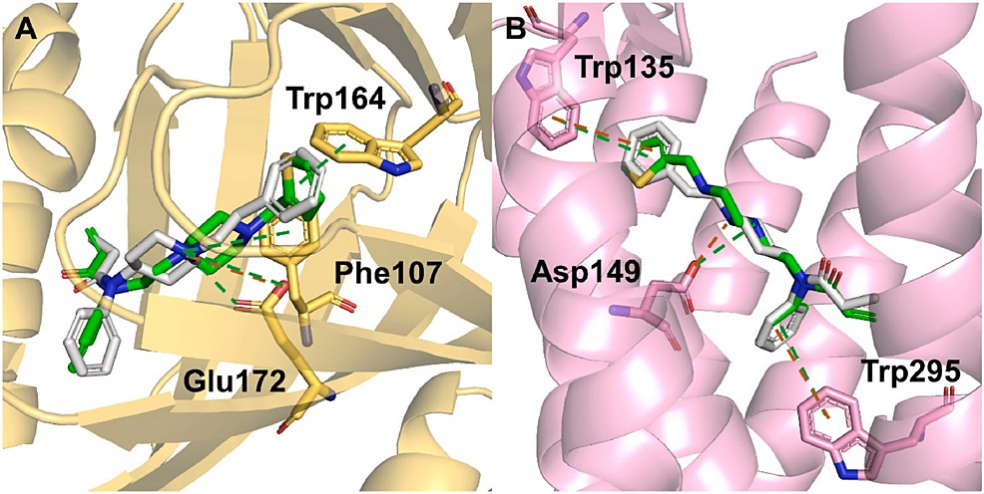
Fitting of compounds into pharmacophores of (A) S1R and (B) MOR.

Most recently, Fan et al. reported on a new series of thiophenpiperazine amide derivatives that display bispecific S1R-antagonist (*K*_i_ = 44.7 nM) + MOR-agonist activity (Figure 1) [24]. The highlighted compound (23) was active (s.c.) in the mouse acetic acid-induced abdominal constriction test and mouse carrageenan-induced inflammatory model. Evaluation of the adverse effect profile of the compound was said to be in progress. 

## Conclusions

The publications summarized herein as examples lead to the conclusion that the strategy of designing bispecific S1R-antagonist/ MOR-agonist compounds provides a hopeful avenue for the development of compounds that produce pain relief against a broad variety of acute and neuropathic clinical pain conditions, with improved safety profile compared to current strong opioid analgesics. Given the recent availability of several bispecific compounds with promising and druggable properties, the efficacy, safety, and clinical utility of such bispecific compounds only await validation in human clinical trials.
